# Exome sequencing of Japanese schizophrenia multiplex families supports the involvement of calcium ion channels

**DOI:** 10.1371/journal.pone.0268321

**Published:** 2022-05-10

**Authors:** Miho Toyama, Yuto Takasaki, Aleksic Branko, Hiroki Kimura, Hidekazu Kato, Yoshihiro Nawa, Itaru Kushima, Kanako Ishizuka, Teppei Shimamura, Tomoo Ogi, Norio Ozaki

**Affiliations:** 1 Department of Psychiatry, Nagoya University Graduate School of Medicine, Nagoya, Aichi, Japan; 2 Division of Systems Biology, Nagoya University Graduate School of Medicine, Nagoya, Aichi, Japan; 3 Department of Genetics, Research Institute of Environmental Medicine, Nagoya University, Nagoya, Japan; 4 Department of Human Genetics and Molecular Biology, Nagoya University Graduate School of Medicine, Nagoya, Japan; 5 Institute for Glyco-core Research (iGCORE), Nagoya University, Nagoya, Japan; University of Colorado Denver - Anschutz Medical Campus, UNITED STATES

## Abstract

**Background:**

Most sequencing studies of schizophrenia (SCZ) have focused on *de novo* genetic variants due to interpretability. However, investigating shared rare variants among patients in the same multiplex family is also important. Relatively large-scale analyses of SCZ multiplex families have been done in Caucasian populations, but whether detected variants are also pathogenic in the Japanese population is unclear because of ethnic differences in rare variants.

**Materials and methods:**

We performed whole-exome sequencing (WES) of 14 Japanese SCZ multiplex families. After quality control and filtering, we identified rare variants shared among affected persons within the same family. A gene ontology (GO) analysis was performed to identify gene categories possibly affected by these candidate variants.

**Results:**

We found 530 variants in 486 genes as potential candidate variants from the 14 SCZ multiplex families examined. The GO analysis demonstrated significant enrichment in calcium channel activity.

**Conclusion:**

This study provides supporting evidence that calcium ion channel activity is involved in SCZ. WES of multiplex families is a potential means of identifying disease-associated rare variants for SCZ.

## Introduction

Schizophrenia (SCZ) is a chronic and severe mental disorder characterized by some combination of hallucinations, delusions, and extremely disordered thinking and behavior that impairs daily functioning [1]. The lifetime prevalence of SCZ is 0.3–0.7%, and the standardized mortality ratio is 2.5 [2, 3]. Both genetic and environmental factors affect the risk for SCZ [4, 5]. Population-based and twin concordance studies indicated that the heritability of SCZ is 60–80%, with only subtle contributions from environmental factors [6, 7].

As genetic factors play a significant role in the etiology of SCZ, many genetic studies of the disease have been conducted. Linkage studies identified multiple genetic risk loci for SCZ; however, these studies were not sufficient to suggest specific causative genes [8]. Genome-wide association studies (GWASs) have identified numerous loci significantly associated with SCZ [9, 10] based on the common disease–common variant hypothesis. However, the effect sizes of the individual single-nucleotide polymorphisms identified in GWASs are too small to explain the high heritability of SCZ demonstrated in cohort studies, which has been designated “missing heritability” [11]. To overcome this problem, it is necessary to consider rare variants such as single-nucleotide variants (SNVs) or copy number variants [12].

The feasibility of exploring disease-associated SNVs has been enhanced due to the advent of whole-genome sequencing (WGS) and whole-exome sequencing (WES) techniques, with the latter providing a more rapid and cost-effective approach for sequencing protein-coding regions across the genome. However, efficiently extracting candidates from a large number of detected mutations is challenging. As such, most of these studies have focused on *de novo* genetic variants or rare variants significantly associated with SCZ due to interpretability [13–16] in terms of their large estimated effect sizes and the possibility of functional validation.

In addition, patients in families with multiple affected members are likely to be enriched in genetic factors that strongly affect the development of SCZ [17]. Therefore, it is also essential to investigate variants shared among patients in order to elucidate the association between transmitted variants and SCZ. Indeed, several sequencing studies have focused on shared variants associated with bipolar disorder [18, 19] and autism spectrum disorder [20, 21] in patients in the same multiplex family. A previous WES study of SCZ multiplex families demonstrated that several loci could potentially affect synaptic plasticity and neurocognitive performance [22] and that variants in genes related to metabotropic glutamate receptor 5 (mGlu5) are more common in affected family members [23].

However, the above-mentioned relatively large-scale sequencing studies have been done in Caucasian populations. The total genetic variation of the Japanese population, however, is considered relatively low [24] compared to that of ethnically diverse populations, in particular Europeans, which can be beneficial in sequencing studies due to decreased allelic diversity [25–27]. Furthermore, rare variants, which were targeted in this study due to their more recent origin, tend to be more geographically clustered and can be population specific, thus potentially revealing new SCZ candidates beyond those identified in studies of Caucasian populations.

We therefore hypothesized that we could identify variants associated with SCZ susceptibility in the Japanese population via analyses of Japanese SCZ multiplex families, even if the sample sizes were small compared with studies of Caucasian populations. Individual rare variants provide limited power for identifying significant trait associations, and thus, multi-variant and/or multi-genic approaches such as gene set enrichment tests are necessary.

To address this hypothesis, a WES study of multiplex families within the Japanese population was conducted to identify disease-associated rare variants or gene sets.

## Materials and methods

### Participants—SCZ multiplex families

**Fig 1:**
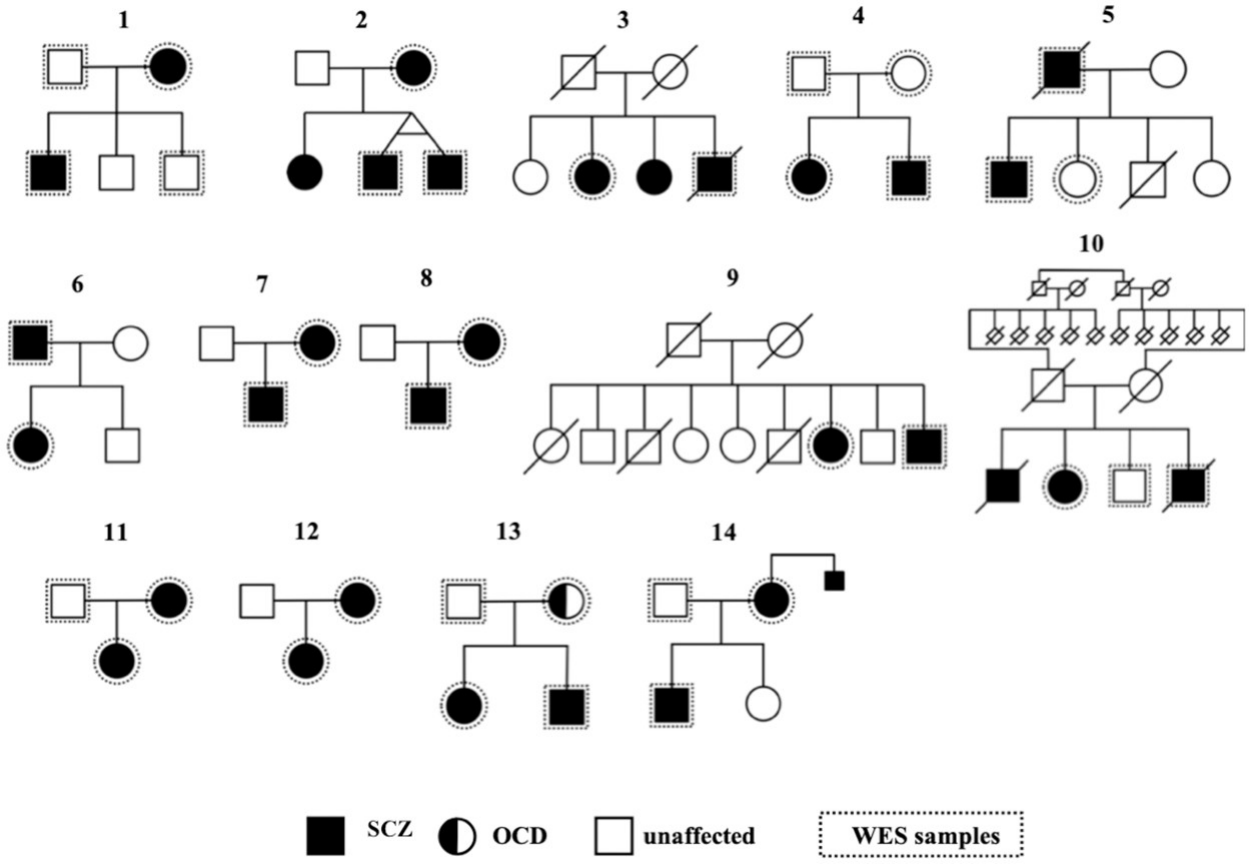
Sequenced samples. Dashed lines indicate individuals who underwent WES. Squares indicate males, and circles indicate females. Shading indicates an affected individual, and slash marks indicate deceased individuals. We performed WES for 29 patients with SCZ, 1 patient with obsessive-compulsive disorder (OCD), and 9 healthy individuals from 14 SCZ multiplex families in Japan.

A multiplex family was defined as a family having more than one member with SCZ. DNA samples were collected from peripheral blood or saliva of 29 patients with SCZ, 1 patient with obsessive-compulsive disorder (OCD), and 9 healthy individuals from 14 SCZ multiplex families in Japan (Fig 1, S1 Table). Among the 14 SCZ multiplex families, pedigree 10 was a consanguineous family. All families were unrelated, lived on the mainland of Japan, and self-identified as Japanese. All patients fulfilled the criteria for SCZ listed in the Diagnostic and Statistical Manual of Mental Disorders–Fifth Edition (DSM-5).

The Ethics Committees of Nagoya University Graduate School of Medicine approved this study, and written informed consent was obtained from all subjects. The study was conducted in accordance with the Helsinki Declaration of 1975 and its later amendments or comparable ethical standards.

### WES

The library was prepared using SureSelect XT Human All Exon V5 (Agilent Technologies). WES was performed on a HiSeq2500 sequencer (Illumina) with paired-end 100-bp reads. WES data reported are available only upon request, as the data contain potentially identifying or sensitive pedigree information. Low-quality reads were excluded using the FASTX-Toolkit, and the remaining reads were mapped to the Human 1kg Reference (GRCh37 + decoy) using the BWA-MEM algorithm. Duplicated reads were removed using Picard. Variants were called using the HaplotypeCaller in the Genome Analysis Toolkit (GATK) [28] and annotated using ANNOVAR [29] with GENCODE Comprehensive gene annotation ver. 19.

### Quality control

To reduce the number of false positives, only SNVs satisfying the following criteria were included: read depth ≥10, genotyping quality ≥20, and alternative allele ratio ≥25%, which was derived from the number of reads with alternative alleles divided by the total number of reads. We also included variants passed through the GATK VQSR filter and not in segmental duplications. Furthermore, using in-house WES data for 1,781 samples, variants with ≥20 detections were excluded to avoid platform-dependent sequencing errors (i.e., false positives). We then performed analyses of relatedness using the—relatedness2 option of vcftools [30] software to confirm the prior information about each multiplex family [31].

### Filtering conditions

To identify pathogenic SNVs and indels, we selected those meeting the following conditions: (1) variants that cause protein alterations; (2) variants located in the splicing site, including synonymous variants detected by ANNOVAR with GENCODE comprehensive gene annotation ver. 19., because synonymous variants that disrupt exonic splice enhancers could be a common cause of genetic disorders [32]; (3) variants with an allele frequency ≤1% in the following databases: 1000 Genome Project (2015 August), total population without psychiatric cohorts in Exome Aggregation Consortium ver. 0.3, Eastern Asian population in Exome Aggregation Consortium ver. 0.3, Human Genetic Variation Database (http://www.hgvd.genome.med.kyoto-u.ac.jp), and Japanese Multi Omics Reference Panel (https://jmorp.megabank.tohoku.ac.jp/); (4) variants shared only among patients (we also selected *de novo* variants only from pedigrees whose parents were unaffected, as pedigrees with affected parents could have shared rare variants between cases); and (5) variants in genes with a percentile residual variation intolerance score (RVIS) [33] (ExAC_0.05 threshold) ≤25% and genes expressed in brain regions based on data from the Human Protein Atlas with normalized expression ≥1, as we assumed that variants occurring in genes highly intolerant of protein alterations and expressed in the brain could be deleterious for biological functions.

For deleterious variants and/or *de novo* or homozygous variants, we manually inspected their calls using Integrated Genomics Viewer [34] ver.2.7.2 and confirmed their exonic functions (change in protein structure and/or function induced by a variant in the exon, such as synonymous, nonsynonymous, or loss of function) using Ensembl genome browser GRCh37 (http://grch37.ensembl.org/index.html).

### Gene ontology (GO) analysis

We performed GO analysis (http://geneontology.org/docs/go-enrichment-analysis/) using Cytoscape and its GeneMANIA plugin [35]. To correct in multiple comparisons, Q-values derived via the Benjamini-Hochberg procedure were used to judge the significance of results. The significance level was set at Q-value <0.05.

## Results

### WES analysis

We performed WES for 14 SCZ multiplex families in Japan (Fig 1). The data were quality checked and filtered using the following criteria: 1) low frequency (an allele frequency ≤1% in the databases), 2) protein-altering (missense and splice site variants), and 3) variants in the intolerant genes (RVIS ≤ 25%) and genes expressed in brain regions. Finally, we identified 525 variants among 481 genes carried by at least two patients in the same family. A summary of the filtered variants is presented in S2 and S3 Tables. Loss-of-function variants among these candidate variants are presented in Table 1. In addition to the 525 variants identified among the 481 genes, we also identified a number of *de novo* variants (Table 2). Therefore, we identified a total of 530 variants among 486 genes as potential multiplex family candidate variants for SCZ.

**Table 1:** Loss-of-function variants.

Pedigree	Genomic position	Function	Gene	cDNA change*	Amino acid change*
1	20:48479568	Frameshift deletion	*SLC9A8*	c.864delA	p.A288fs
3	19:56000877	Frameshift insertion	*SSC5D*	c.210dupG	p.P70fs
6	10:26785321	Splicing	*APBB1IP*	c.160+1G>C	-
7	19:33167791	Frameshift deletion	*RGS9BP*	c.622_643del	p.P208fs
8	1:60306057	Frameshift deletion	*HOOK1*	c.615delT	p.D205fs
8	16:3165404	Stopgain	*ZNF205*	c.G106T	p.E36X
9	2:84932821	Stopgain	*DNAH6*	c.C8677T	p.R2893X
10	2:80085187	Stopgain	*CTNNA2*	c.C347A	p.S116X
10	18:6898571	Stoploss	*ARHGAP28*	c.T2167C	p.X723Q
10	7:12384097	Splicing	*VWDE*	c.3887-2->T	-
11	17:18150343	Stopgain	*FLII*	c.G2700A	p.W900X
11	19:33134477	Splicing	*ANKRD27*	c.585+1G>A	-
14	4:186357227	Frameshift insertion	*C4orf47*	c.348_349insAT	p.T116fs
14	8:29043913	Frameshift deletion	*KIF13B*	c.392_393del	p.F131fs
14	16:74694876	Frameshift deletion	*RFWD3*	c.465_472del	p.V155fs

Genomic positions correspond to the NCBI37/hg19 build;

*Longest transcription.

**Table 2:** Homozygous (hemizygous) and *de novo* variants.

Pedigree	Sample ID	Genomic position	Function	Gene symbol	G_Change	AA_Change	MAF				
							ExAC_ALL	ExAC_EAS	1000G_ALL	HGVD	jMorp
*De novo*											
4	N0674	12:2224447	Nonsynonymous SNV	*CACNA1C*	c.C107T	p.A36V	0	0	0	0	0
4	N0674	19:14046846	Nonsynonymous SNV	*PODNL1*	c.G352A	p.E118K	0	0	0	0	0
4	N0673	19:39993536	Nonsynonymous SNV	*DLL3*	c.G491T	p.R164L	0	0	0	0	0
13	N1010	2:10582149	Nonsynonymous SNV	*ODC1*	c.C902T	p.T301M	0.000022	0.0002	0	0	0
13	N1010	9:139964552	In-frame deletion	*SAPCD2*	c.359_361del	p.120_121del	0	0	0	0	0
Homozygous											
10	N0688, N0689	3:1363404	Nonsynonymous SNV	*CNTN6*	c.T832G	p.S278A	0.000011	0.0002	0	0.0016	0.002
Hemizygous											
13	N0812, N0813 (N1010)	X:43662605	Nonsynonymous SNV	*MAOB*	c.C326T	p.P109L	0.0004	0	0.00052	0.00042	0

Abbreviations: MAF, minor allele frequency; ExAC, exome aggregation consortium; EAS, Eastern Asian; 1000G, 1000 Genome Project; HGVD, Human Genetic Variation Database; jMorp, Japanese Multi-Omics Reference Panel. Genomic position based on NCBI build GRCh 37/hg19.

Note: N1010 is the mother of N0812 and N0813, who were affected with obsessive compulsive disorder with heterozygous variants in the X chromosome.

Of the 14 pedigrees examined, seven (pedigrees 1, 4, 5, 10, 11, 13, and 14) included control samples. We selected 199 variants in 190 genes found in these seven pedigrees as “strict-filtered” for further analysis, as we sequenced both affected and unaffected family members in these pedigrees to exclude variants carried by healthy individuals.

### GO analysis

To identify gene categories that could be affected by the identified candidate variants, we performed GO analysis. Significantly enriched categories (Q-value <0.05) are shown in Table 3. We tested the enrichment of the 199 variants identified in the 190 “strict-filtered” genes (pedigrees 1, 4, 5, 10, 11, 13, and 14) and found significant enrichment of genes associated with calcium channel activity (Q = 0.032). A similar result was also observed for the 530 variants identified in 486 genes that were derived from whole pedigrees in this study (Q = 0.011).

**Table 3:** GO analysis results.

GO ID	GO term	Q-value	Coverage	*Gene*
*Genes from 7 pedigrees*, *including control samples (strict-filtered)*				
GO:0005262	Calcium channel activity	0.032	7/62	*CACNA1C*, *RYR2*, *CACNB1*, *CACNA1E*, *LOXHD1*, *RYR1*, *CACNA1G*
GO:0015085	Calcium ion transmembrane transporter activity	0.033	7/73	*CACNA1C*, *RYR2*, *CACNB1*, *CACNA1E*, *LOXHD1*, *RYR1*, *CACNA1G*
GO:0005245	Voltage-gated calcium channel activity	0.033	5/26	*CACNA1C*, *CACNB1*, *CACNA1E*, *RYR1*, *CACNA1G*
*Genes from all 14 pedigrees in this study*				
GO:0042391	Regulation of membrane potential	0.0023	18/165	*WWP2*, *LRRK2*, *SHANK1*, *KCNH7*, *IFI6*, *MAPK8IP2*,
				*KCNQ1*, *DSC2*, *ANK3*, *ACSBG1*, *CHRNA4*, *CACNA1E*,
				*SKI*, *RYR2*, *NRXN1*, *CACNA1G*, *ANK2*, *AKAP6*
GO:0043269	Regulation of ion transport	0.011	19/219	*DIAPH1*, *WWP2*, *CAPN3*, *STIM2*, *CNKSR3*, *CACNA1C*,
				*SHANK1*, *WNK2*, *AKT1*, *HOMER1*, *MAPK8IP2*, *KCNQ1*, *CAMK2G*, *ANK3*, *CHRNA4*, *RYR2*, *NRXN1*, *ANK2*, *AKAP6*
GO:0005262	Calcium channel activity	0.011	10/62	*RYR1*, *ITPR1*, *STIM2*, *CACNB1*, *CACNA1C*, *LOXHD1*,
				*RYR3*, *CACNA1E*, *RYR2*, *CACNA1G*
GO:0015085	Calcium ion transmembrane transporter activity	0.011	11/73	*RYR1*, *ITPR2*, *STIM2*, *CACNB1*, *CACNA1C*, *LOXHD1*,
				*RYR3*, *CACNA1E*, *RYR2*, *CACNA1G*
GO:0034765	Regulation of ion transmembrane transport	0.026	13/119	*WWP2*, *STIM2*, *SHANK1*, *WNK2*, *AKT1*, *HOMER1*,
				*MAPK8IP2*, *KCNQ1*, *ANK3*, *RYR2*, *NRXN1*, *ANK2*, *AKAP6*
GO:0072509	Divalent inorganic cation transmembrane transporter activity	0.026	11/88	*RYR1*, *ITPR1*, *STIM2*, *CACNB1*, *CACNA1C*, *LOXHD1*,
				*RYR3*, *ITPR2*, *CACNA1E*, *RYR2*, *CACNA1G*
GO:0010959	Regulation of metal ion transport	0.026	14/143	*DIAPH1*, *WWP2*, *CAPN3*, *STIM2*, *CNKSR3*, *CACNA1C*,
				*WNK2*, *HOMER1*, *KCNQ1*, *CAMK2G*, *ANK3*, *RYR2*,
				*ANK2*, *AKAP6*
GO:0032409	Regulation of transporter activity	0.026	12/105	*WWP2*, *STIM2*, *SHANK1*, *WNK2*, *HOMER1*, *KMT2A*,
				*MAPK8IP2*, *ANK3*, *RYR2*, *NRXN1*, *ANK2*, *AKAP6*
GO:0034762	Regulation of transmembrane transport	0.026	13/126	*WWP2*, *STIM2*, *SHANK1*, *WNK2*, *AKT1*, *HOMER1*,
				*MAPK8IP2*, *KCNQ1*, *ANK3*, *RYR2*, *NRXN1*, *ANK2*, *AKAP6*
GO:0032411	Positive regulation of transporter activity	0.026	7/34	*STIM2*, *WNK2*, *KMT2A*, *ANK3*, *RYR2*, *ANK2*, *AKAP6*
GO:0032412	Regulation of ion transmembrane transporter activity	0.026	11/92	*WWP2*, *STIM2*, *SHANK1*, *WNK2*, *HOMER1*, *MAPK8IP2*,
				*ANK3*, *RYR2*, *NRXN1*, *ANK2*, *AKAP6*
GO:0022898	Regulation of transmembrane transporter activity	0.026	11/94	*WWP2*, *STIM2*, *SHANK1*, *WNK2*, *HOMER1*, *MAPK8IP2*,
				*ANK3*, *RYR2*, *NRXN1*, *ANK2*, *AKAP6*
GO:0051235	Maintenance of location	0.041	15/176	*ABCA1*, *CER1*, *RYR1*, *NFKBIE*, *ITPR1*, *OSBPL11*, *DAG1*,
				*ENPP1*, *LATS1*, *SHANK1*, *NFKB1*, *TLN1*, *ANK3*, *SYNE1*,
				*RYR2*
GO:2000021	Regulation of ion homeostasis	0.044	11/100	*RYR1*, *DIAPH1*, *CAPN3*, *ITPR1*, *CACNA1C*, *WNK2*, *IFI6*,
				*ANK3*, *RYR2*, *ANK2*, *AKAP6*

Abbreviations: GO, Gene Ontology.

Note: GO analysis was performed with 190 *genes from 7 pedigrees*, *including control samples (strict-filtered)* and 486 genes from all 14 pedigrees in this study. The genes used in the GO analysis are listed in S4 Table.

## Discussion

This is the first report of a WES analysis of Japanese SCZ multiplex families. After WES analysis and filtering of 14 SCZ multiplex families, we selected variants shared only among patients (we also selected *de novo* variants only from pedigrees whose parents were unaffected, as pedigrees with affected parents could have shared rare variants between cases), and we identified a total of 530 SCZ candidate SNVs and indels among 486 genes. In an *in silico* analysis involving 530 SCZ candidate SNVs and indels among 486 genes, we demonstrated that many candidate variants were located in genes related to calcium ion channels that have also been reported as involved in the pathophysiology of SCZ, as demonstrated by common variants from genome-wide association analyses [10] and rare variants from whole-genome CNV/SNV analyses [16, 36]. For example, *CACNA1C*, which was identified as a candidate gene in this study, was also identified as a SCZ susceptibility gene in a GWAS [10] as well as exome [16] and whole-genome CNV [36] analyses.

The 486 candidate genes from 530 SCZ candidate variants identified in our present study were enriched in calcium channel activity–related genes as the most enriched GO terms (Table 3), although there could have been enrichment of calcium channel–related GO terms among the top 25% RVISs and brain-expressed genes. Voltage-gated calcium channels are widely distributed in all parts of the brain. They are critical for mediating intracellular Ca^2+^ influx, which results in transmitter release from pre-synaptic endings, thereby affecting neuronal excitability and synaptic plasticity and playing a role in neurodevelopmental disorders such as SCZ [37]. On the other hand, in this study, we did not observe any GO enrichment in the SCZ-associated genes reported in two previous WES studies of SCZ multiplex families [22, 23] such as *AMACR*, a gene involved in fatty acid metabolism and previously implicated in SCZ [22], and genes related to the metabotropic glutamate receptor 5 (Table 3) [23].

Among the 530 candidate variants identified, we detected a male carrier of a hemizygous variant in *MAOB* (located on the X chromosome) and one homozygous variant in *CNTN6* in a pedigree with consanguineous marriage (Table 2). The p.P109L variant in *MAOB* identified in family 13 was previously reported in a male SCZ patient as being inherited from his heterozygous mother [38]. This variant may cause a change in the structure of the protein’s binding site to the mitochondrial membrane [39]. We also identified this hemizygous variant in a male SCZ patient segregated from his heterozygous mother with OCD (Fig 1). Interestingly, *MAOB* is suggested as being associated with OCD [40].

The homozygous SNV in *CNTN6* shared among affected members of pedigree 10 with consanguineous marriage is also interesting, as several recent reports have identified *CNTN6* as a candidate gene involved in neurodevelopmental disorders, including SCZ [41, 42]. SNVs in *CNTN6* are significantly associated with Autism spectrum disorder in particular [43]. *CNTN6* encodes contactin 6, which plays a role in neuronal cell adhesion and promotes neurite outgrowth in sensory-motor neuronal pathways [44]. The homozygous variant we identified is located in the immunoglobulin C2–type domain (IGC domain), which mediates interactions with contactin-binding partners such as protein tyrosine phosphatase receptor–gamma (Ptptg), which plays a role in the molecular basis of neurodevelopmental functions [44]. Moreover, among the 486 genes examined in the present study, three genes (*RBM12*, *NRXN1*, *AKT1*) are registered in the OMIM (https://www.ncbi.nlm.nih.gov/omim) database as being associated with susceptibility to SCZ.

Although our results provide some support for the calcium channel–associated hypothesis, there are still limitations to our study. First, our sample size was small for an investigation of the burden and/or transmission of rare and *de novo* protein-altering mutations between affected and non-affected individuals, and thus, our results could be misleading. Second, we could not evaluate clinical phenotypes, especially within unaffected families. Therefore, we could not evaluate the clinical phenotype of unaffected family members with discovered variants associated with susceptibility to SCZ, such as *MAOB* and *CNTN6*. Furthermore, in this study, we could only elucidate minimal variant information for each pedigree due to data availability constraints. In future multiplex familial studies, it will be important to evaluate in detail the relationship between variants and phenotype within the same family. Third, due to the study’s small sample size, the experiments could have generated false-positive results, and strict filtering could have led to some false-negative results. The different strategies we used in the study to narrow down candidate variants likely limited false positives, however. More importantly, SCZ is not a monogenic disorder, so filtering is not necessary to identify only a few pathogenic variants; identifying disease-associated pathways would also be a useful approach to elucidate the mechanism of the disorder.

## Conclusion

In conclusion, using WES analysis of 14 Japanese multiplex families, we identified a number of rare variants segregated in SCZ patients. Our results provide support for the hypothesis that calcium channel activity is related to the development of SCZ. Analyzing a larger sample size of multiplex families could confirm these results and provide additional information regarding the aspects of this disease.

## Supporting information

S1 Table Information regarding samples used in this study. (DOCX)

S2 Table Summary of filtered variants. (DOCX)

S3 Table Information of filtered variants in this study. (XLSX)

S4 Table List of genes used in the GO analysis. (XLSX)
