# Supplementary material for: Exome sequencing of Japanese schizophrenia multiplex families supports the involvement of calcium ion channels
Source: PLoS One. 2022 May 10;17(5):e0268321. doi: 10.1371/journal.pone.0268321 (PMC9089874; doi:10.1371/journal.pone.0268321)
Supplement: S1 Table — (DOCX) [file pone.0268321.s001.docx]

**Table S1. Information regarding samples used in this study**

|  | SCZ |
| --- | --- |
| Family Number of families | 14 |
| Number of Samples | 39 |
| Probands (%) | 29 (74%) |
| Healthy subjects (%) (including 1 OCD mother) | 10 (26%) |
|  |  |
| All |  |
| Males (%) | 21 (54%) |
| Mean age (SD) | 54 (16.6 years) |
|  |  |
| Probands |  |
| Males (%) | 14 (48%) |
| Mean age (SD) | 51 (15.9 years) |

Abbreviations: OCD, obsessive compulsive disorder; SCZ, schizophrenia; SD, standard deviation.
