# Supplementary material for: Exome sequencing of Japanese schizophrenia multiplex families supports the involvement of calcium ion channels
Source: PLoS One. 2022 May 10;17(5):e0268321. doi: 10.1371/journal.pone.0268321 (PMC9089874; doi:10.1371/journal.pone.0268321)
Supplement: S2 Table — (DOCX) [file pone.0268321.s002.docx]

**Table S2. Summary of filtered variants**

| Category | heterozygous  (14 families) | heterozygous  (7 families: Strict-filtered) |
| --- | --- | --- |
| Splicing(≦±2) | 3 | 2 |
| Stopgain | 4 | 2 |
| Stoploss | 1 | 1 |
| Frameshift deletion | 5 | 3 |
| Frameshift insertion | 2 | 1 |
| LoF | 15 | 9 |
| Nonsynonymous SNV | 449 | 170 |
| Nonframeshift deletion | 10 | 6 |
| Nonframeshift insertion | 5 | 0 |
| Synonymous(exonic;splicing) | 14 | 4 |
| Splicing(＞±2) | 32 | 10 |
| Sum | 525 | 199 |

Abbreviations: LoF, loss of function; SNV, single nucleotide variant.
